# The discovery of the hydrogen bond from p-Nitrothiophenol by Raman spectroscopy: Guideline for the thioalcohol molecule recognition tool

**DOI:** 10.1038/srep31981

**Published:** 2016-09-23

**Authors:** Yun Ling, Wen Chang Xie, Guo Kun Liu, Run Wen Yan, De Yin Wu, Jing Tang

**Affiliations:** 1 Key Laboratory of Analysis and Detection Technology for Food Safety, Ministry of Education, College of Chemistry, Fuzhou University, Fuzhou 350108, China; 2 State Key Laboratory of Marine Environmental Science, College of the Environment and Ecology, Xiamen University, Xiamen, 361002, China; 3 State Key Laboratory of Physical Chemistry of Solid Surfaces, Department of Chemistry, College of Chemistry and Chemical Engineering, Xiamen University, Xiamen, 361005, China

## Abstract

Inter- and intra- molecular hydrogen bonding plays important role in determining molecular structure, physical and chemical properties, which may be easily ignored for molecules with a non-typical hydrogen bonding structure. We demonstrated in this paper that the hydrogen bonding is responsible for the different Raman spectra in solid and solution states of p-Nitrothiophenol (PNTP). The consistence of the theoretical calculation and experiment reveals that the intermolecular hydrogen bonding yields an octatomic ring structure 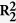 (8) of PNTP in the solid state, confirmed by the characteristic S-H---O stretching vibration mode at 2550 cm^−1^; when it comes to the solution state, the breakage of hydrogen bond of S-H---O induced the S-H stretching vibration at 2590 cm^−1^. Our findings may provide a simple and fast method for identifying the intermolecular hydrogen bonding.

Hydrogen bonding is ubiquitous in nature and central to the structure and biological functions^1^,^2^,^3^,^4^,^5^. Experimental and theoretical spectroscopic studies of weakly bonded intermolecular complexes provide a wealth of information on the structure and dynamics of such species and define a starting point for a detailed understanding of various macroscopic phenomena. Raman spectroscopy, as one of the powerful vibrational spectroscopy, has been applied to studying inter- and intra- molecular interactions by analyzing the line profiles and wavenumber shifts of selected vibrational Raman bands^6^,^7^.

Recently, PNTP^8^,^9^,^10^ is widely used as a probe molecule to understand the electrochemical^11^,^12^,^13^,^14^,^15^,^16^ and photochemical reaction mechanisms^17^,^18^,^19^,^20^,^21^,^22^,^23^,^24^,^25^,^26^,^27^,^28^,^29^,^30^,^31^,^32^,^33^. The self-assembly of PNTP onto rough silver or gold surfaces has been characterized by surface-enhanced Raman spectroscopy (SERS) with the disappearance of the S-H stretching band at ca. 2550 cm^−1 ^12,17,18,19,34,35. By zooming in the normal Raman spectrum of PNTP solid in the 2500–2640 cm^−1^ region (as shown in Fig. 1a inset), we can observe a weak peak at ca. 2590 cm^−1^, whose Raman intensity is around 5 times less than that of the traditionally assigned S-H stretching band at 2550 cm^−1^. However, the density functional theory (DFT) calculation with the Gaussian 09 software showed that the 2594 cm^−1^ but not 2550 cm^−1^ peak is from the S-H stretching vibrational of PNTP, and the 2550 cm^−1^ peak is non-observable (seeing in Fig. 1b inset). We also simulated the Raman spectra of PNTP adsorbed on gold and silver surfaces, which there are no peaks in the Raman spectra region 2500–2640 cm^−1^ of the S-H stretching vibration for Au_5_-PNTP and Ag_5_-PNTP (simulation as shown in Fig. S1).

## Results and Discussion

The difference between the theoretical calculation and the experiment lies that the theoretical one is on the basis of the free molecule without any interference from surrounding molecules, whereas the experimental result is obtained from the solid state sample. It is well-known that the Raman vibration is ultra-sensitive to the molecular structure, therefore, the inconsistence between calculation and experiment might be ignited from the strong intermolecular interaction between two neighbour PNTP molecules in solid state, considering the disulfide bonding^36^ between the two S-H or hydrogen-bonding^37^ between S-H and N-O groups, respectively. By so far, there is no report related to the PNTP crystal structure.

In order to figure out the origin of the two peaks of ca. 2550 cm^−1^ and 2590 cm^−1^ observed in the S-H stretching vibration region, we simulated the Raman spectra of 4-Nitrophenyl disulphide (NPDS), the disulfate structure of PNTP (as shown in Fig. 2a), and 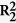
**(8)** structure^3^, the hydrogen bonding dimer of two PNTP molecules using density functional theory calculations (seeing in Fig. 2b).

No surprisingly, we can’t observe these two peaks of ca. 2550 cm^−1^ and 2590 cm^−1^ in the simulated Raman spectrum of NPDS, due to the disappearance of S-H bond via the formation of disulfate. Instead, the peak at ca. 1084 cm^−1^ of PNTP is split to two peaks at ca. 1059 and 1099 cm^−1^ of NPDS, which is confirmed by the experimental Raman spectrum of NPDS (seeing Supplementary in Fig. S2).

**Figure 1:**
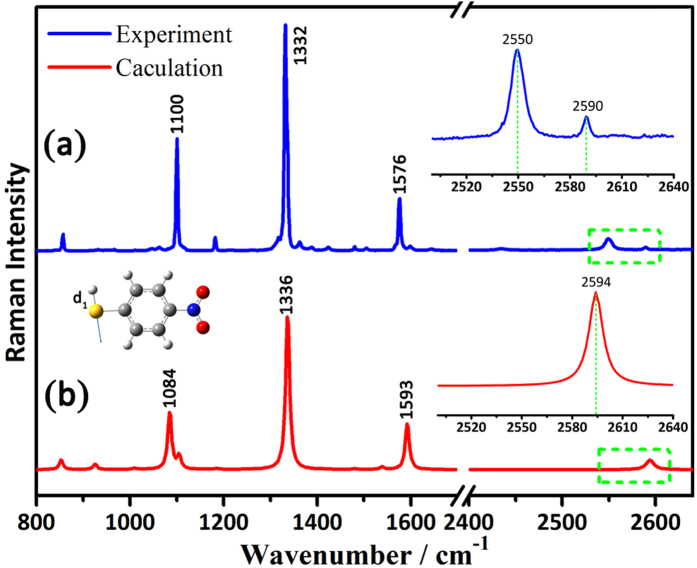
Experimental (**a**) and Theoretical (**b**) Raman spectra of PNTP. Inset: zooming in the region 2500–2640 cm^−1^.

**Figure 2:**
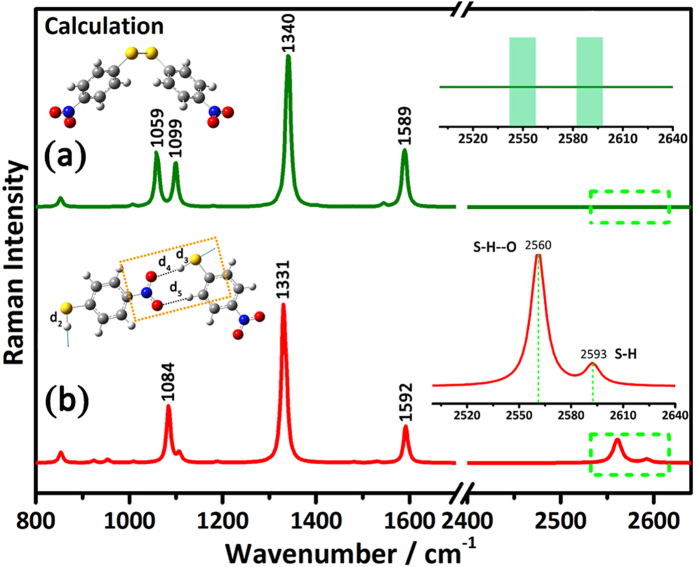
Theoretical Raman spectra and molecular structure and vibrational modes. (**a**) Theoretical Raman spectra of NPDS. (**b**) Theoretical Raman spectra of two PNTP molecules with hydrogen bonds. Inset: molecular structure and zooming in the region 2500–2640 cm^−1^.

When it comes to the case of the hydrogen-bonding dimer system as shown in Fig. 2b, the two peaks at 2560 cm^−1^ and 2593 cm^−1^ were clearly displayed, very similar to the two peaks observed experimentally. Furthermore, the relative Raman intensity of the 2560 cm^−1^ peak to the 2593 cm^−1^ is 5–6 times, a value almost identical to the experimental result shown in Fig. 1a. Whereas hydrogen bonding has a negligible effect on the other main characteristic Raman peaks of PNTP, such as 1084 cm^−1^ (νC-S), 1336 cm^−1^ (νNO_2_) and 1593 cm^−1^(νC-C).

Taking a look at the molecular structure of this dimer shown in Fig. 2b inset, we find that an octatomic ring 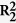 **(8)** is formed with the hydrogen bonding between S-H and N-O groups of the two neighbour PNTP molecules. Compared to the S-H free of hydrogen bonding (d_2_, 1.348 Å), the interaction between S and H atoms for the d_3_ bond (1.351 Å) is weaker due to the formation of the S-H**---**O structure, which will induce the redshift of S-H vibration from 2593 cm^−1^ to 2560 cm^−1^. Meanwhile, the formation of the hydrogen-bonding octatomic ring greatly increases the Raman scattering cross section of the d_3_ bond, which results in a much stronger Raman intensity of d_3_ than d_2_ (More detailed explanation in supplementary Fig. S3). These phenomenons may be explained with proton transfer mechanism^38^,^39^,^40^,^41^. Therefore, the Raman spectrum obtained from the solid PNTP is not from the PNTP itself, but the dimer structure shown in the insert of Fig. 2b.

**Figure 3:**
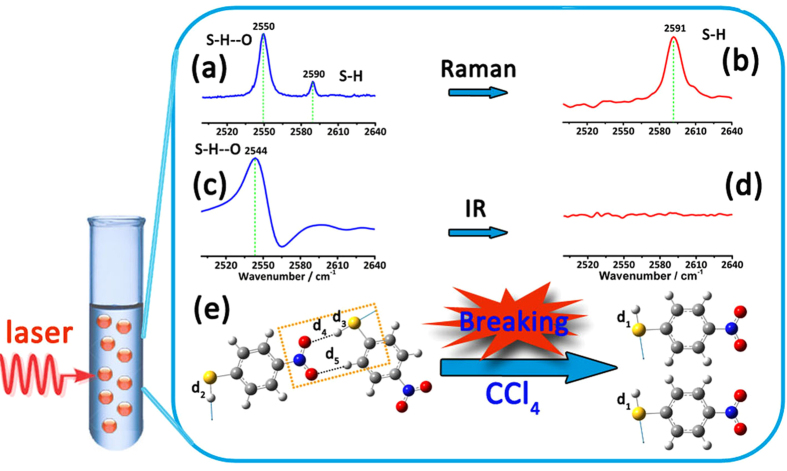
(**a**) Normal Raman spectra of the PNTP solid. (**b**) Normal Raman spectra of PNTP dissolved in CCl_4_. (**c**) IR spectra of the PNTP solid. (**d**) IR spectra of PNTP dissolved in CCl_4_. (**e**) Schematic illustration of hydrogen bond structure and breakage into two PNTP molecules.

Considering the hydrogen bonding is very sensitive to the environment and could be easily broken or formed with the solvation effect^42^,^43^, we dissolved the PNTP in tetrachloromethane (20 mM) to obtain the normal Raman spectrum in Fig. 3b. One Raman band at 2591 cm^−1^ is clearly displayed, which can be assigned to the S-H stretching vibration of the free PNTP molecule and is in good agreement with the calculated value of 2594 cm^−1^ (shown in Fig. 3e). The appearance of 2550 cm^−1^ band (shown in Fig. 3a) is originated from the association of molecules through the hydrogen bonding. PNTP dissolved in the CCl_4_ induced the blue shift of the three main characteristic Raman peaks at 1103 cm^−1^ (νC-S), 1344 cm^−1^ (νNO_2_) and 1583 cm^−1^ (νC-C) comparing with that of the PNTP solid one (seeing Supplementary in Fig. S4). Besides, Fig. 3c,d also show the infrared spectra of PNTP in the solid state and in CCl_4_ solution, respectively. One could clearly observe that the disappearance of the ν_S-H--O_ peak at the 2544 cm^−1^ (More experimental and theoretical spectra seeing Supplementary in Figs S5 and S6) after the PNTP molecule being dissolved in CCl_4_, which is further confirmed the hydrogen bonding of PNTP in the solid state. Similar solvation effect was observed in in other solvents, such as dichloromethane (seeing Supplementary in Fig. S7).

## Conclusion

In summary, hydrogen bonding between two PNTP molecules dimer was investigated by Raman spectroscopy and DFT calculations. The Raman spectra sensitively captured partial structure changes of the molecule. Structures and vibrational modes for pure PNTP and hydrogen-bonded 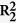 **(8)** complex with the stoichiometric ratio 1:1 were calculated. Both calculations and experiments show that 2550 cm^−1^ can be assigned to the S-H stretching vibration of the S-H**---**O structure, and 2590 cm^−1^ is assigned to the S-H stretching vibration free of hydrogen bonding. The observation not only indicates that conclusions stemmed from S-H stretching band of the PNTP molecule in the literatures might need to be reinterpreted, but also may provide new insight for the widely investigated interfacial chemical reaction of PNTP^26^,^27^,^28^,^29^,^30^,^31^,^32^,^33^ under external field by Surface-enhanced Raman spectroscopy (SERS) or Tip-enhanced Raman spectroscopy (TERS). Besides, the current work provides a guideline for the thioalcohol molecules recognition tool, a simple and fast method for identifying the intermolecular hydrogen bonding.

## Methods

PNTP was purchased from Aldrich. NPDS was purchased from Aladdin. CH_2_Cl_2_ was purchased from Sinopharm Chemical Reagent Co. Ltd. CCl_4_ was purchased from Tianjin ZhiYuan Reagent Co. Ltd. KBr was purchased from Tianjin FuChen Chemical Reagent Co. Ltd. All reagents were used without further purification.

The IR spectra of 20 mM PNTP in CCl_4_ was recorded at room temperature with an ATR-FTIR spectrometer (Nicolet iS50). The IR spectra of PNTP solid with KBr was obtained at room temperature using an FT-IR spectrometer (Nicolet 6700).

All the Raman signals were obtained at room temperature using a confocal Raman microscopy system (inVia Renishaw, UK) with a 532-nm laser for excitation. The Raman spectral resolution was 1 cm^−1^. The diameter of the light spot was ~1.5 μm and the laser excitation power was 0.15 mW for measuring the Raman spectrum of solid state of PNTP and NPDS. The diameter of the light spot was ~6.5 μm and the laser excitation power was 10.5 mW for measuring the Raman spectrum of 20 mM PNTP in CCl_4_ and CH_2_Cl_2_. All spectra were recorded using an accumulation time of 10 s.

The theoretical calculations of the molecular Raman spectra and IR spectra and their vibrational modes were performed with the Gaussian 09 software using density functional theory, the B3LYP functional^44^,^45^, and the 6–311+G(p, d) level of theory.

## Additional Information

**How to cite this article**: Ling, Y. *et al*. The discovery of the hydrogen bond from p-Nitrothiophenol by Raman spectroscopy: Guideline for the thioalcohol molecule recognition tool. *Sci. Rep.* **6**, 31981; doi: 10.1038/srep31981 (2016).

## Supplementary Material

Supplementary Information
