# Supplementary material for: The discovery of the hydrogen bond from p-Nitrothiophenol by Raman spectroscopy: Guideline for the thioalcohol molecule recognition tool
Source: Sci Rep. 2016 Sep 23;6:31981. doi: 10.1038/srep31981 (PMC5034243; doi:10.1038/srep31981)
Supplement: Supplementary Information [file srep31981-s1.pdf]

Supplementary information

The discovery of the hydrogen bond from p-nitrothiophenol by Raman spectroscopy: Guideline for the thioalcohol molecule recognition tool

Yun Ling [1], Wen Chang Xie[1], Guo Kun Liu[2,*], Run Wen Yan[3], De Yin Wu[3], and Jing Tang[1,*]

[1]Key Laboratory of Analysis and Detection Technology for Food Safety, Ministry of Education, College of Chemistry, Fuzhou University, Fuzhou 350108, China

[2]State Key Laboratory of Marine Environmental Science, College of the Environment and Ecology, Xiamen University, Xiamen 361002, China

[3]State Key Laboratory of Physical Chemistry of Solid Surfaces, Department of Chemistry, College of Chemistry and Chemical Engineering, Xiamen University, Xiamen 361005, China.

* Corresponding authors:

Tel./Fax: + 86-591-22866165

E-mail addresses: jingtang@fzu.edu.cn (J. Tang), guokunliu@xmu.edu.cn (G. Liu)

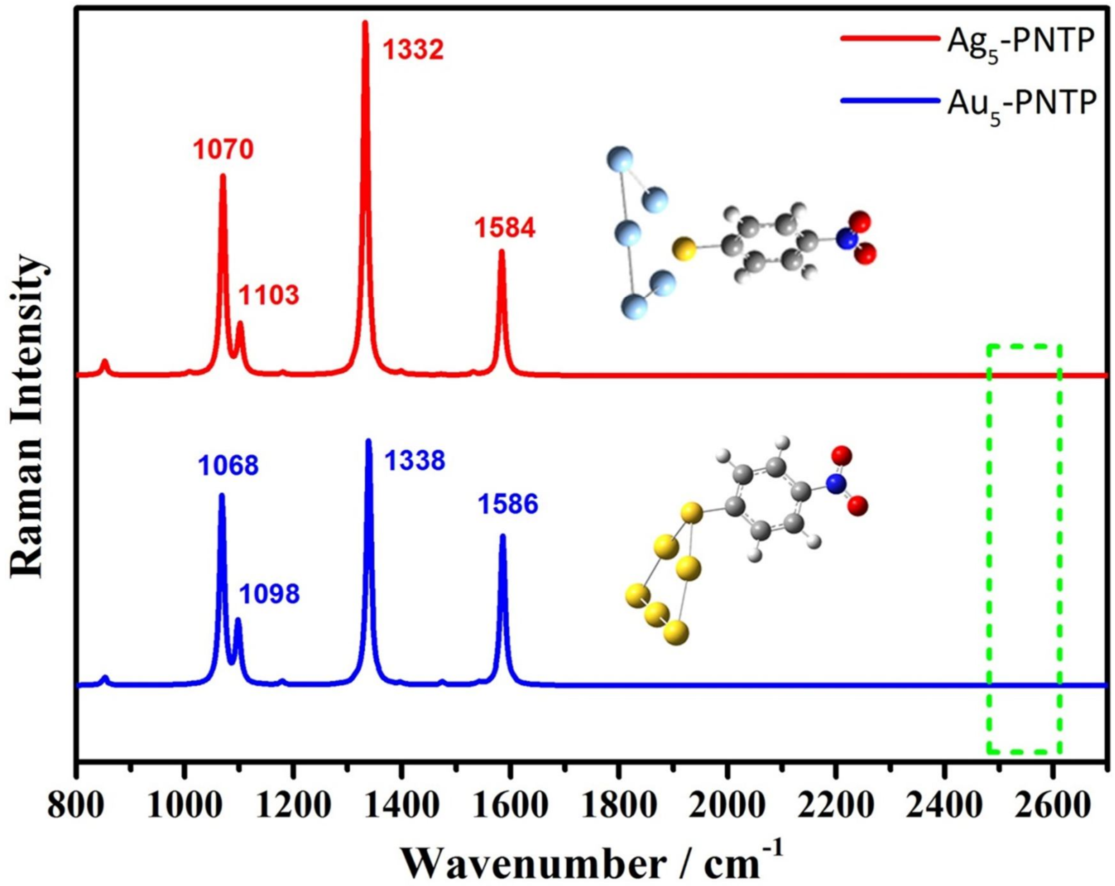

Figure S1. Simulated Raman spectra of PNTP adsorbed on gold and silver surfaces.

The metallic cluster model was employed to investigate the Raman spectra of PNTP on gold and silver surfaces. Figure S1 shows that there are no peaks in the Raman spectra region 2500-2640 cm^{-1} of the S-H stretching vibration for Au_5-PNTP and Ag_5-PNTP.

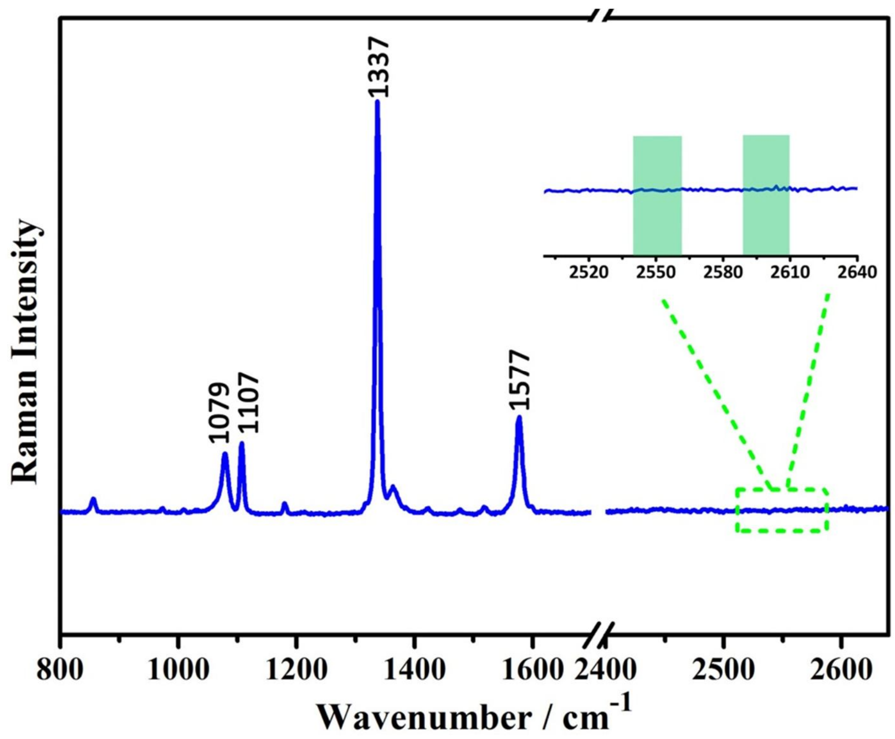

Figure S2. Experimental normal Raman spectra of NPDS. Inset: zooming in the region 2500-2640 cm^{-1}

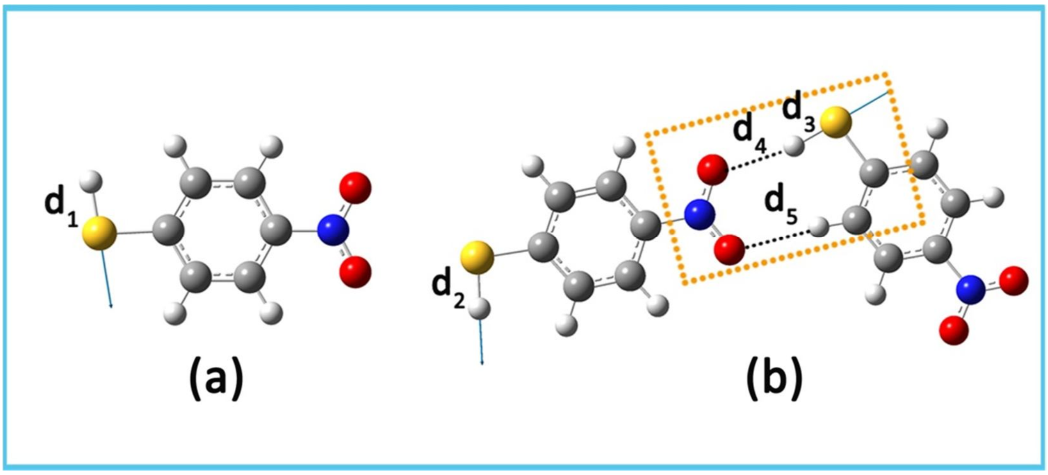

Figure S3. Vibrational modes of the free PNTP molecule (a); Vibrational modes and structure of hydrogen-bonding dimer with two PNTP molecules (b)

Figure S3b shows that the two PNTP molecules lie in nearly the same plane. An octatomic ring is formed by two hydrogen bonds and both bonds are not equivalent. The hydrogen bond d_4 between the O atom on the NO_2 and the H atom on the S-H group is shorter (S─H---O, 2.29 Å) than the hydrogen d_5 with the O atom and C-H

(C—H---O, 2.50 Å). The hydrogen bond is more linear with the inter-bond angle C—H---O ≈ 178°, whereas the S—H---O angle is ca 167°. In the structure, the S-H bond d_3 length is 1.351 Å and it is longer compared to the S-H bond d_2 length (1.348 Å) which is virtually identical to the S-H bond d_1 length (1.347 Å) of PNTP single molecule. Hydrogen bond lengthens the S-H bond d_3 length.

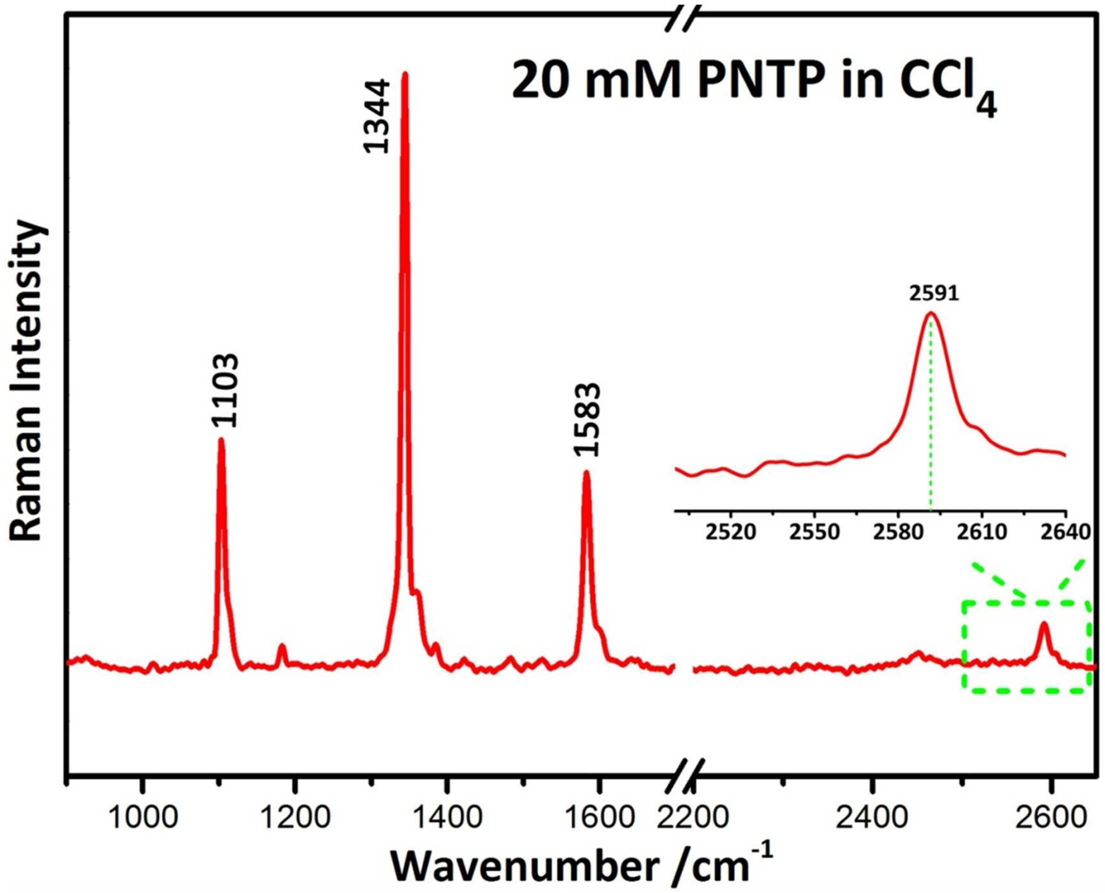

Figure S4. Normal Raman spectra of PNTP dissolved in CCl_4, which is a subtraction spectra result of PNTP and CCl_4 using the math program provided by Labspec software (Horiba/JY). Inset: zooming in the region 2500-2640 cm^{-1}

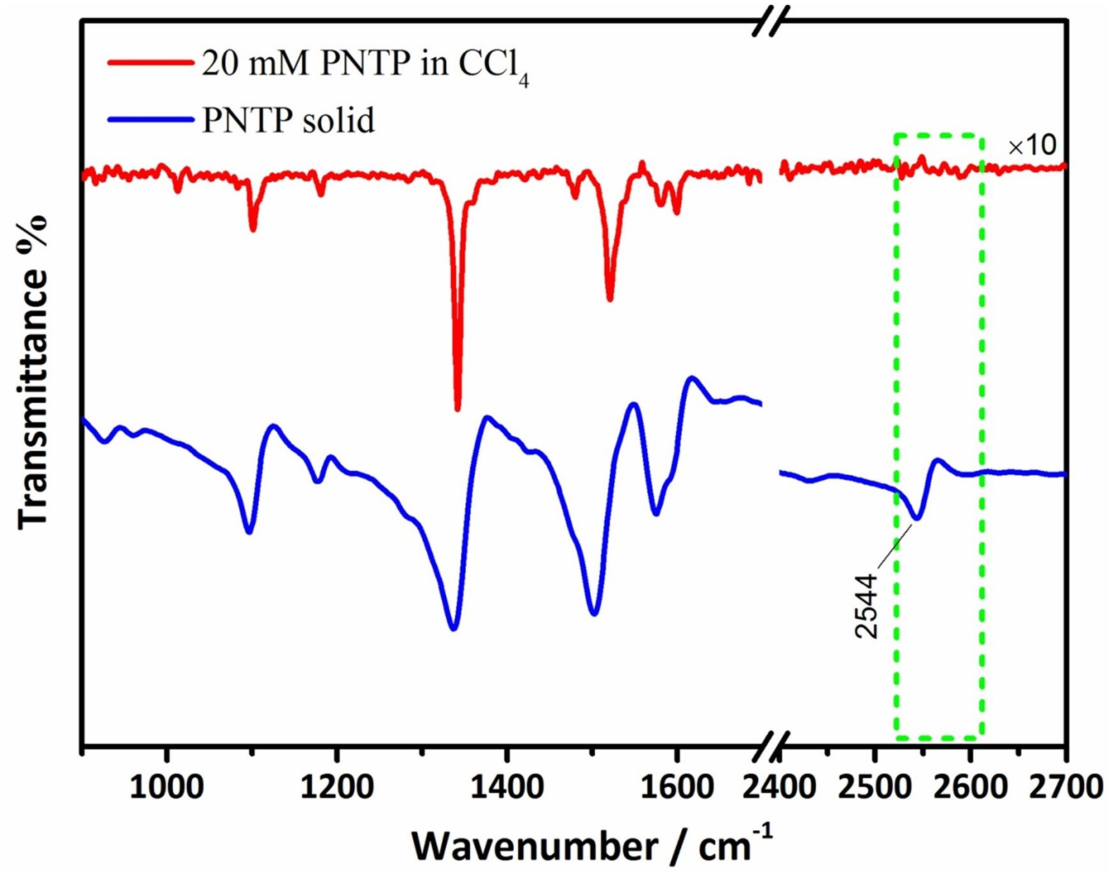

Figure S5. IR spectra of PNTP solid and 20 mM PNTP dissolved in CCl_4

The 2544cm^{-1} can be assigned to S-H---O stretching vibration from PNTP solid, and there is no peak in the 2500-2640 cm^{-1} region from 20 mM PNTP in CCl_4.

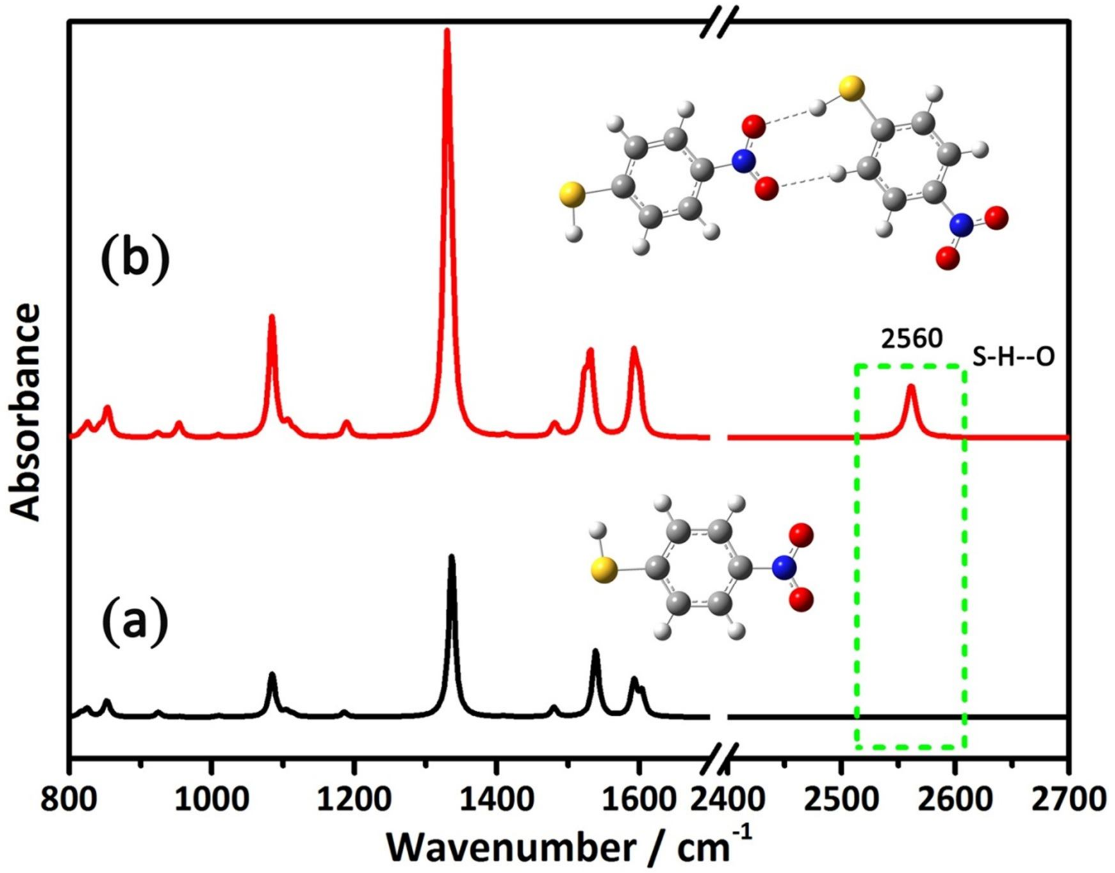

Figure S6. Simulated IR spectra of single molecule of PNTP (a) and two molecules of PNTP with hydrogen bonds(b).

The 2560cm^{-1} can be assigned to S-H---O stretching vibration from the Fig. S6b, and there is no peak in the 2500-2640 cm^{-1} region from the Fig. S6a.

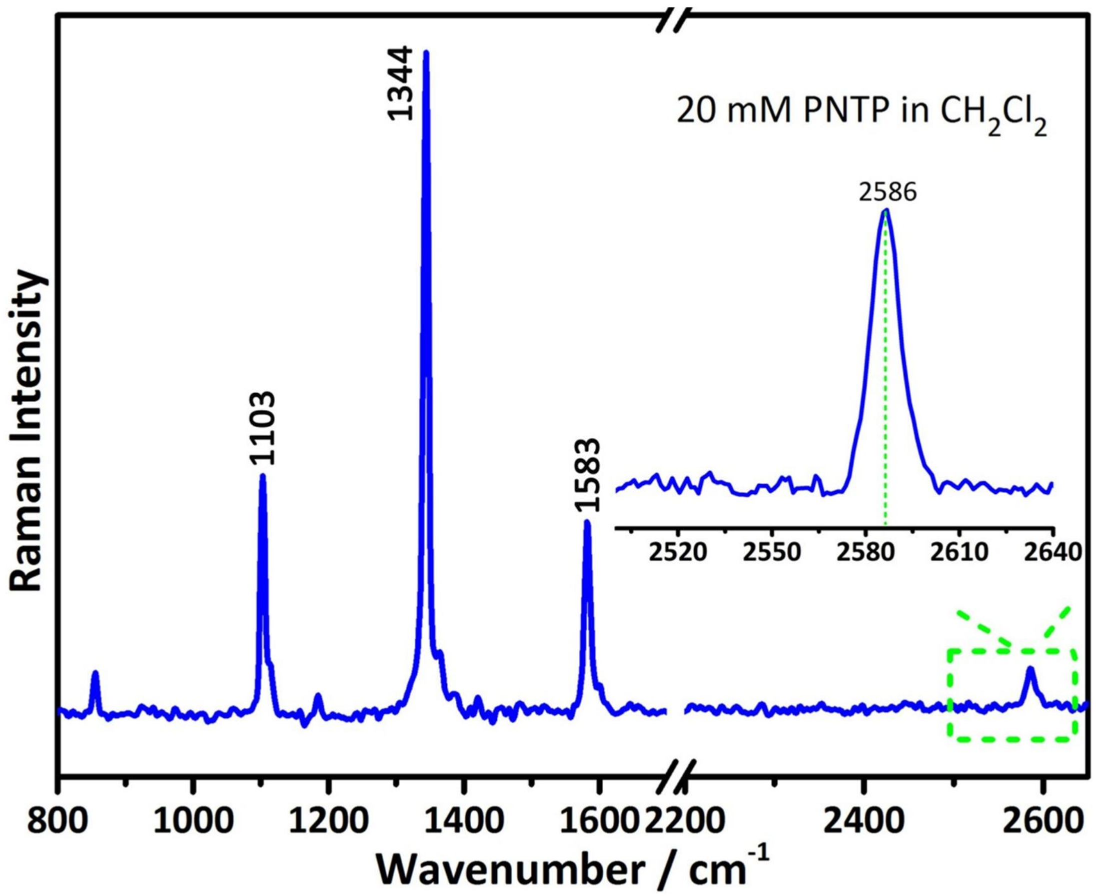

Figure S7. Normal Raman spectra of PNTP dissolved in CH_2Cl_2, which is a subtraction spectra result of PNTP and CH_2Cl_2 using the math program provided by Labspec software (Horiba/JY). Inset: zooming in the region 2500-2640 cm^{-1}
